# Is joint hypermobility linked to self-reported non-recovery from COVID-19? Case–control evidence from the British COVID Symptom Study Biobank

**DOI:** 10.1136/bmjph-2023-000478

**Published:** 2024-02-20

**Authors:** Jessica A Eccles, Dorina Cadar, Lisa Quadt, Alan J Hakim, Nicholas Gall, Covid Symptom Survey Biobank Consortium, Vicky Bowyer, Nathan Cheetham, Claire J Steves, Hugo D Critchley, Kevin A Davies

**Affiliations:** 1 Department of Clinical Neuroscience, Brighton and Sussex Medical School, Brighton, UK; 2 Neurodevelopmental Service, Sussex Partnership NHS Foundation Trust, Worthing, UK; 3 Division of Medicine, School of Medicine, Penn State University, University Park, Pennsylvania, USA; 4 Department of Neurology, National Hospital for Neurology and Neurosurgery, Queen Square, University College London Hospitals NHS Foundation Trust, London, UK; 5 Department of Neurocardiology, King’s College Hospital NHS Foundation Trust, London, UK; 6 Department of Twin Research and Genetic Epidemiology, King's College London, London, UK; 7 Department of Ageing and Health, Guys and St Thomas’s NHS Foundation Trust, London, UK; 8 Department of Clinical and Experimental Medicine, Brighton and Sussex Medical School, Brighton, UK

**Keywords:** COVID-19, mass screening, SARS-CoV-2, translational science, biomedical, Post-Acute COVID-19 Syndrome

## Abstract

**Objectives:**

This study sought to explore whether generalised joint hypermobility (GJH, a common marker of variant connective tissue) was a risk factor for self-reported non-recovery from COVID-19 infection.

**Design:**

Prospective observational study.

**Setting:**

COVID Symptom Study Biobank (https://cssbiobank.com/) UK

**Participants:**

Participants were surveyed in August 2022. 3064 (81.4%) reported at least one infection with COVID-19. These individuals self-reported on recovery and completed a self-report questionnaire to detect GJH (Hakim and Grahame 5-part questionnaire, 5PQ).

**Main outcome measures:**

The primary outcome was the presence of self-reported non-recovery from COVID-19 infection at the time of the survey. Additional outcomes included scores on 5PQ and self-reported fatigue level (Chalder Fatigue Scale).

**Results:**

The presence of GJH was not specifically associated with reported COVID-19 infection risk per se. However, it was significantly associated with non-recovery from COVID-19 (OR 1.43 (95% CI 1.20 to 1.70)). This association remained after sequential models adjusting for age, sex, ethnic group, education level and index of multiple deprivation (OR 1.33 (95% CI 1.10 to 1.61)) and further adjustment for vaccination status and number of vaccinations (OR 1.33 (95% CI 1.10 to 1.60)). Additionally, including in a model adjusting for all covariates, hypermobility significantly predicted higher fatigue levels (B=0.95, SE=0.25, t=3.77, SE, p=0.002). Fatigue levels mediated the link between GJH and non-recovery from COVID-19 (estimate of indirect effect=0.18, 95% bootstrapped CI 0.08 to 0.29).

**Conclusions:**

Individuals with GJH were approximately 30% more likely not to have recovered fully from COVID-19 infection at the time of the questionnaire, and this predicted the fatigue level. This observation is clinically important through its potential impact for understanding and identifying sub-phenotypes of long COVID for screening and personalised targeted interventions. More generally, greater awareness of GJH and its extra-articular associations is needed for effective patient stratification and implementation of personalised medicine.

**WHAT IS ALREADY KNOWN ON THIS TOPIC**

- Long COVID represents one of the greatest clinical challenges and public health burdens of a generation.
- In addition to demographic factors such as increased age, the likelihood of developing long COVID appears to be increased by the presence of a pre-existing activity-limiting health condition or disability; for example, fibromyalgia, irritable bowel syndrome, migraine, allergies, anxiety, depression and back pain are among a number of conditions identified as raising the risk of long COVID. These risk factors have all independently been associated with a common variant of connective tissue—namely, joint hypermobility.
- Currently no studies have explicitly explored the potential unifying link of generalised joint hypermobility as a risk factor for not recovering fully from COVID-19 infection.

**WHAT THIS STUDY ADDS**

- This study suggests that the presence of generalised joint hypermobility may be a risk factor for not recovering fully from COVID-19 and predicts greater fatigue levels.
- This has significant implications for further research, clinical practice and public health, including precision healthcare approaches.

**HOW THIS STUDY MIGHT AFFECT RESEARCH, PRACTICE OR POLICY**

- This study highlights the need for research on predisposing factors and comorbidities on risk factors, particularly as they relate to variant connective tissue (joint hypermobility)
- The study also highlights the need for stratified personalised healthcare for patients and the need to inform and plan policy and multidisciplinary service provision for those with long COVID and related conditions.

## Introduction

### What is the clinical problem?

 The COVID-19 pandemic and the burden of subsequent limited recovery from COVID-19^1^ present some of the greatest clinical challenges of a generation. Such individuals not fully recovered from COVID-19 may self-identify under the collective patient-advocated term ‘long COVID’ and/or meet one of the various clinical definitions created to describe persistent symptoms.^2^ National data from March 2023 indicate that around 3% of the UK population had not recovered fully from COVID-19 infection. The most common associated symptoms are fatigue (72%), difficulty concentrating (51%), muscle aches/pain (49%) and shortness of breath (48%).^3^ However, more than 200 symptoms, expressed across multiple organ systems, are associated with delayed recovery following acute COVID-19 infection.^1^ A history of infection with COVID-19 is now commonplace,^4^ so a precise and mechanistic understanding of factors that predispose to enduring symptoms and limit recovery will help design and deliver effective healthcare to improve the quality of life of millions of affected individuals worldwide. Evidently, the presentation of long COVID is heterogeneous.^5^ These distinct profiles need further characterisation in order to design personalised care and to target effective treatment across populations and age groups.

### What do we know about long COVID at this point?

In addition to demographic factors, notably female sex, the likelihood of developing long COVID appears to be increased by the presence of pre-existing activity-limiting health conditions or disabilities.^3^ Fibromyalgia, irritable bowel syndrome, migraine, anxiety, depression and back pain^6^ are among a number of conditions identified as raising the risk of long COVID. However, other studies on long COVID have failed to find specific associations, particularly in relation to pre-existing affective disorders.^7^ There is growing awareness of the symptomatic overlap with myalgic encephalomyelitis or chronic fatigue syndrome (ME/CFS), in which viral infection is often implicated as a trigger. Indeed, such research may help elucidate the aetiology of long COVID.^8^ Moreover, cardiovascular autonomic dysfunction, particularly postural orthostatic tachycardia syndrome (POTS), may be precipitated by COVID-19 and expressed in long COVID.^9^ POTS is a chronic and often disabling disorder characterised by orthostatic intolerance with an excessive increase in heart rate without hypotension during upright posture. Patients often experience a constellation of other typical symptoms overlapping with long COVID including fatigue and exercise intolerance, and it is established that the onset of POTS may be precipitated by immunological stressors. A variety of pathophysiologies are involved in the abnormal postural tachycardia response; however, the precise aetiology of the syndrome is incompletely understood and is undoubtedly multifaceted.^10^ Indeed, the presence of POTS is considered to be a major sub-phenotype of long COVID, with an estimated prevalence of 30–75% in symptomatic patients, depending on methodology.^11^,^13^ These conditions (POTS, ME/CFS) are archetypes of seemingly complex poorly-understood multisystem illnesses, alongside hypermobility spectrum disorder (HSD)^14^ and Ehlers–Danlos syndrome, which we discuss further below.^15^ The mechanisms and pathobiology behind the association of long COVID with these multiple co-occurring conditions are currently poorly understood. However, convergent biological mechanisms, including dysregulated autonomic, inflammatory and metabolic processes, are increasingly implicated in the expression and maintenance of long COVID.^1 16 17^ Understanding and managing such complexity can be challenging to clinicians who may resort to heuristic classifications such as ‘functional’ disorder. As a consequence, many patients report stigmatisation and can wait years for a diagnosis to access appropriately targeted and potentially effective treatment.^18^ Similar perceptions appear to be emerging in relation to long COVID.^19 20^ This may compromise the planning and delivery of cost-effective and timely therapy, both at the level of the individual patients and the level of healthcare service provision.

There is growing interest in how variant connective tissue (often recognised by the presence of generalised joint hypermobility (GJH)) may represent a common constitutional factor predisposing to such complex multisystem conditions and disorders (see box 1). GJH is a characteristic marker of hereditary disorders of connective tissue, which ultimately compromises a matrix of proteins that includes collagens, elastins, fibrillins and tenascins. Joint hypermobility is typically more common in females and declines with age. GJH itself is not necessarily a medical problem, but certain clinical phenotypes, notably hypermobile Ehlers–Danlos syndrome (hEDS; previously known as EDS hypermobility type/EDS type-III) and hypermobility spectrum disorder (HSD) are associated with clinically significant issues, including chronic disabling fatigue and dysautonomia.^14^ Around 20% of the UK population fulfil the criteria for GJH as an indicator of variant connective tissue structure.^21^ However, less clear is whether individuals with GJH are predisposed to COVID-19 infection and impaired recovery.

Box 1 **Clinical phenomena associated with symptomatic joint hypermobility (HSD/EDS)**

**Strong association with symptomatic joint hypermobility (HSD/EDS):**

- Chronic pain (including headache, migraine and orofacial pain due to temporomandibular joint disease; visceral pain; musculoskeletal pain including oligoarticular/polyarticular pain, and neuropathic pain).
- Chronic fatigue sufficiently disabling to interfere with day-to-day function.
- Functional gastrointestinal disorders (Rome IV criteria), including slow gastrointestinal transit.
- Postural orthostatic tachycardia syndrome, orthostatic hypotension, orthostatic intolerance.
- Neurological manifestations (in adults): atlanto-axial/cranio-cervical joint instability, Chiari 1 malformation, small fibre neuropathy/disease, local anaesthetic resistance.
- Developmental coordination disorder.
- Anxiety disorders, phobias, depression.

**Evolving evidence of clinical association with symptomatic joint hypermobility (HSD/EDS), however this remains an area with clinical and scientific uncertainties:**

- Neurally-mediated hypotension.
- Neurological manifestations (in children and adolescents).
- Interstitial cystitis.
- Underactive and hyperactive bladder.
- Dysmenorrhoea or dysmenorrhagia.
- Attention deficit/hyperactivity disorder and autism.
- Immune dysregulation, intolerances and allergies.

Adapted and reproduced with permission of the hEDS/HSD Working Group, The International Consortium on the Ehlers-Danlos syndromes and Hypermobility Spectrum Disorders.

### Aim of this study

The aim of this study was to determine, from the UK COVID Symptom Study Biobank (CSSB), if the presence of GJH is associated with an increased risk of not recovering fully from infection with COVID-19.

## Methods

### Public and patient involvement

The research question, choice of hypermobility measures and dissemination strategy was informed by patient and public involvement including priorities, experience and preferences of those living with long COVID. This was crystalised at a POTS UK Long COVID masterclass in 2022 and has been supported by ongoing involvement.

The CSSB is supported by patient and public involvement in order to determine opinions and perspectives of those affected by COVID during the development and running of their research. To help with this, the CSSB has Volunteer Advisory Panels (VAPs). VAPs each consist of 12 representative participants from across the UK. The panels were formed in summer 2021 and member terms are 2 years. Examples of how VAP members advise CSSB research include giving feedback about the feasibility of research plans and suggesting perspectives for researchers to consider when planning investigations and considering results.

### Participants

Data were extracted from the UK CSSB, which was linked to demographic data, SARS-CoV-2 test results and symptom assessment data from the COVID Symptom Study app, a mobile health application developed by ZOE Global with input from physicians and scientists at King’s College London, Massachusetts General Hospital, and Lund and Uppsala Universities.^22 23^

### Outcomes

GJH was determined using the 5-part Hakim and Grahame self-report questionnaire (5PQ), where a score of ≥2 indicates GJH (see box 2 for details).^24^ Recovery from COVID-19 was operationalised from a dichotomous self-reported response to the question “Thinking about the last or only episode of COVID-19 you have had, have you now recovered and are back to normal?” These questions were surveyed as part of the CSSB in August 2022. Fatigue level was quantified by the Chalder Fatigue Scale.

Box 2 **The 5-part Hakim and Grahame Questionnaire (5PQ) for defining generalised joint hypermobility**

1. Can you now (or could you ever) place your hands flat on the floor without bending your knees?
2. Can you now (or could you ever) bend your thumb to touch your forearm?
3. As a child did you amuse your friends by contorting your body into strange shapes OR could you do the splits?
4. As a child or teenager did your shoulder or kneecap dislocate on more than one occasion?
5. Do you consider yourself double-jointed?

Endorsement of two or more questions suggests generalised joint hypermobility.

### Statistical analysis

Binary logistic regression was used to determine if the presence of GJH was a predictor of non-recovery from COVID-19. Age, sex, ethnic group, socioeconomic status (index of multiple deprivation quintile) and education level, vaccination status and number of vaccinations were entered as potential confounders in sequential models. Linear regression was used to explore associations between GJH and fatigue levels, with covariates as above. Mediation analyses were conducted according to the method of Hayes. In such a model a variable is considered to be a likely mediator of the relationship between two independent variables if the bootstrapped CI of the estimate of the indirect effect of the mediator variable on that relationship does not cross zero.^25^ All data were analysed using IBM SPSS Statistics 28.

## Results

In this sample, 3064 (81.4%) reported at least one infection with COVID-19. Of these individuals, data regarding self-reported recovery were available for 2854 participants (2331 (81.7%) female; 2767 (97.4%) white), with a mean age of 57.5 years (range 21–89). A full demographic breakdown is shown in table 1. Reporting at least one COVID-19 infection was not significantly associated with GJH.

**Table 1:** Demographics of sample

	Overall	COVID recovered	COVID non-recovered
Number (%)	2854	1940 (67.8%)	914 (32%)
Female, n (%)	2331 (81.7%)	1556 (80.2%)	775 (84.8%)
White, n (%)	2767 (97.0%)	1889 (97.4%)	878 (96.1%)
Age, mean (SD)	57.50 (10.45)	57.95 (10.48)	56.54 (10.31)
Generalised joint hypermobility, n (%)	708 (24.8%)	439 (22.6%)	269 (29.4%)

Of the 914 participants (32.0%) who reported not having recovered fully from COVID-19 infection, 269 patients (254 (29.4%) female) had GJH. In the fully recovered group, 439 of 1940 patients (22.6%, 400 female) had GJH.

GJH was significantly associated with not having recovered from COVID-19 (Model 1; B=0.355, p<0.001) with OR 1.43 (95% CI 1.20 to 1.70). This association remained significant after sequential corrections for age and sex (Model 2), ethnic group, socioeconomic status and education level (Model 3) (see online supplemental table 1 for details).

In the final model (Model 4, online supplemental table 2) also adjusting for vaccination status and number, GJH remained significantly associated with not having recovered from COVID (B=0.282, p=0.003) with OR 1.33 (95% CI 1.10 to 1.60).

**Figure 1:**
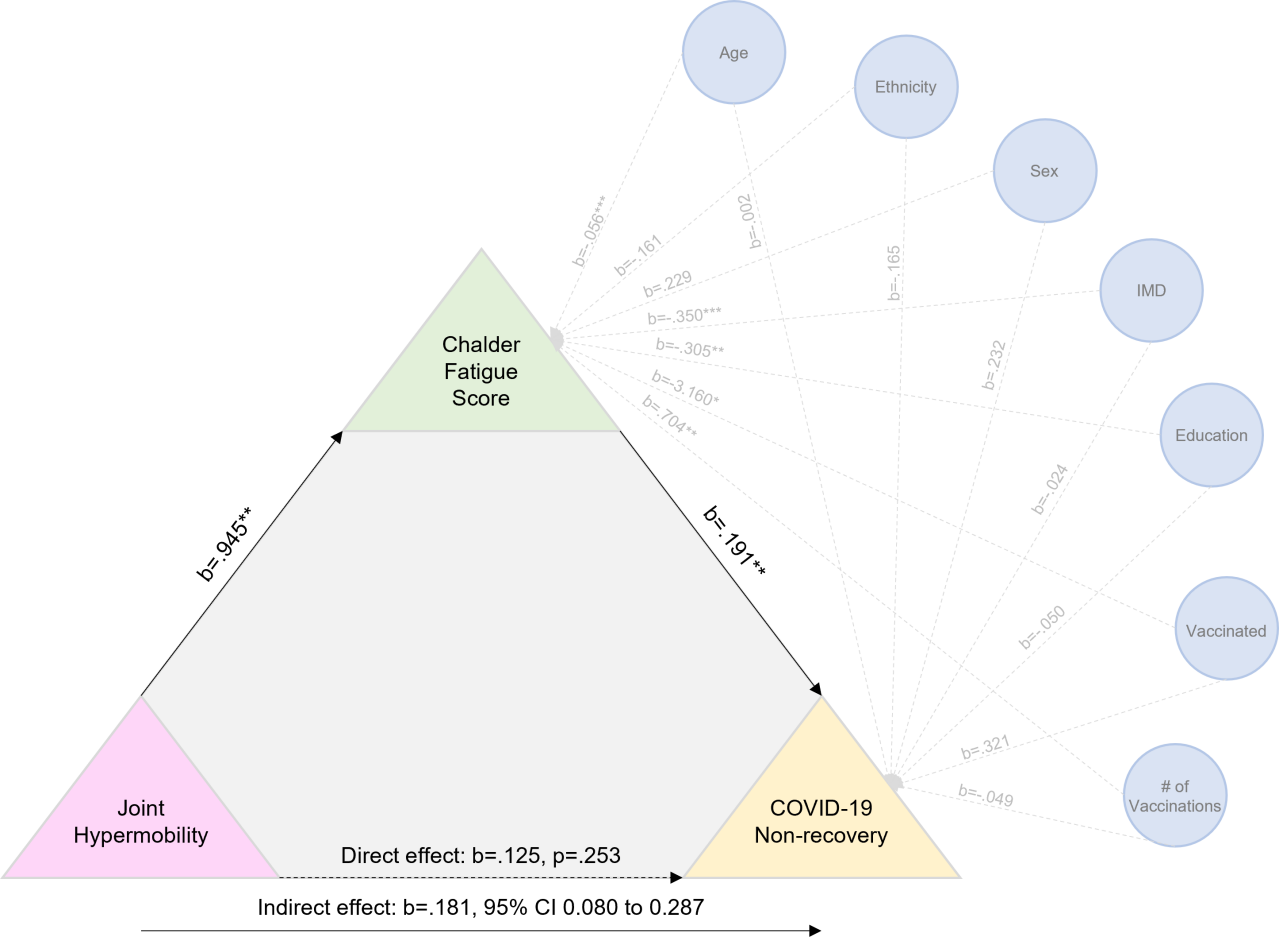
Mediation model showing likely mediating link of fatigue in the relationship between generalised joint hypermobility and not recovering from COVID-19.

Furthermore, in a model adjusting for all covariates, GJH significantly predicted higher fatigue levels (B=0.95, SE=0.25, t=3.77, SE, p=0.002). Moreover, fatigue levels mediated the link between GJH and non-recovery from COVID-19 (estimate of indirect effect=0.18, bootstrapped 95% CI do not cross zero, 0.08 to 0.29) (see figure 1 for details of mediation model).

## Discussion

In this large population-based study, an individual with GJH (an index of variant connective tissue structure) was approximately 30% more likely not to have recovered after initial COVID-19 infection. This observation is clinically important through its potential impact for understanding and identifying sub-phenotypes of long COVID for screening and personalised targeted interventions.

To our knowledge, this is the first published study to directly report on the association between recovery from COVID-19 and a common variant of connective tissue (identifiable from GJH). Furthermore, we propose that this linked through the fatigue level, suggesting that GJH may represent a subtype of those with persistent symptoms from COVID-19. Our study was conducted in a relatively large sample and included comparison with those who had recovered from COVID-19 infection. Our findings are in common with a recent survey exploring GJH in long COVID alone, which found an increased risk of joint, muscle/nerve pain and cognitive fatigue symptoms.^26^

GJH is frequently under-reported and clinically significant subtypes are underdiagnosed. However, this manifestation of a variant of connective tissue is a significant but maybe a commonly overlooked contributing factor to medical consultations.^27^ It is likely a constitutional feature predisposing to a number of multisystem features. In a recent study of patients with clinically significant pain and fatigue (fibromyalgia and ME/CFS) we observed that the majority (81%) also had symptomatic joint hypermobility, yet less than a quarter of these individuals had received a previous diagnosis.^28^

A major strength of the present study is our use of a widely used and well-validated screening tool to detect GJH in those who are likely unaware of their own joint hypermobility status. The conventional way to identify GJH is by a physical examination, scored according to the Beighton score.^29^ This is both a strength and a limitation; however, Beighton scoring can be time consuming in clinical practice and is often unfeasible in large population-based studies. The 5PQ was developed using the Beighton scale as a reference standard, with high sensitivity and specificity of 71–84% and 77–89%, respectively.^30^

A limitation of this study is our inclusion of a predominantly female and white sample. Also, the lack of a stringent fixed definition of long COVID (beyond not recovering in full from COVID-19 infection) represents a potential general weakness, but it aligns with the ecological and pragmatic approach taken by the UK Office of National Statistics. Furthermore, the present analysis does not include or correct for possible confounders such as duration of symptoms and coronavirus variant type, or pre-existing conditions such as fibromyalgia, a condition in which nocioplastic pain and associated symptoms such as fatigue and brain fog are characteristic. A similar issue has recently been raised in the context of patients with pre-existing inflammatory conditions in which similar difficulties arise when interpreting the individual contribution of COVID-related morbidity in the context of pre-existing symptomatology related to underlying inflammatory rheumatic disease.^31^

Our finding that not recovering fully from COVID-19, alongside female sex, is associated with a constitutional feature (GJH) predisposing to premorbid health conditions is consistent with other larger studies and meta-analyses.^1 32^ An examination of over one million individual electronic healthcare records in the UK concluded that delayed recovery from COVID-19 was associated with poor pre-pandemic general and mental health.^33^ A recent review of what is understood about long COVID highlighted potential developmental and immune risk factors including attention deficit hyperactivity disorder, chronic urticaria and allergic rhinitis. This review also noted the potential mechanistic and phenotypic overlap with illnesses including ME/CFS.^1^ An analysis of risk factors for long COVID, in which 384 137 individuals were followed up for a minimum of 3 months following acute COVID-19 infection, found the strongest associations with long COVID included fibromyalgia (adjusted Hazard Ratio (aHR) 1.37, 95% CI 1.28 to 1.47), anxiety (aHR 1.35, 95% CI 1.31 to 1.39), depression (aHR 1.31, 95% CI 1.27 to 1.34), migraine (aHR 1.26, 95% CI 1.22 to 1.30), multiple sclerosis (aHR 1.26, 95% CI 1.03 to 1.53), coeliac disease (aHR 1.25, 95% CI 1.09 to 1.43) and learning disability (aHR 1.24, 95% CI 1.11 to 1.40).^6^ GJH, although advantageous in some contexts, is an index of variant connective tissues throughout bodily systems and is consequently associated with a number of co-occurring physical and psychological health conditions^34^ (see box 1); joint hypermobility is increasingly implicated as a unifying trait that may underlie all of the conditions noted above.^2835^,^41^ Moreover, it is highly associated^42^ with POTS, a manifestation of dysautonomia and one of the most frequently identified sequelae of long COVID.^11^

As previously suggested, long COVID is likely a heterogenous entity encompassing and intersecting immunological, inflammatory, autonomic, respiratory and cardiovascular processes that lead to distinct profiles of symptoms affecting body and brain.^5^ There are probably multifactorial aetiologies and therefore no single treatment strategy for long COVID. However, these results suggest further exploration of whether GJH is linked to a particular phenotype or subtype of those not recovering fully from COVID-19, including long COVID.

GJH may be regarded by many clinicians as an incidental finding or simple variation of normality, where hyperextensible joints may almost represent a clinical ‘red herring’. However, the presence of joint laxity provides an important clue to differences in connective tissue composition that can affect multiple bodily systems. Our findings suggest an increased risk of not recovering fully from COVID-19 for those with joint hypermobility. If this is ultimately associated with a distinct phenotype, this will facilitate screening and personalised treatments—for example, targeting autonomic dysfunction.

This study offers fresh and novel insights into understanding the potential links between pre-existing conditions and long COVID. Confirming these findings in larger and more diverse samples will permit detailed characterisation of those features of GJH (and variant connectivity tissue) that predict particular long COVID phenotypes, with mechanistic implications. Any such study should focus on the contribution of pre-existing conditions as potential risk factors, particularly those associated with multiple bodily symptoms including nocioplastic pain and autonomic dysfunction. More generally, greater awareness of joint hypermobility and of its extra-articular associations is needed for effective patient stratification and implementation of personalised medicine at the level of the individual and society.

## supplementary material

10.1136/bmjph-2023-000478 online supplemental file 1

## Data Availability

Data are available upon reasonable request from the UK COVID Symptom Study Biobank (CCSB) and are not publicly available.
