# Supplementary material for: Is joint hypermobility linked to self-reported non-recovery from COVID-19? Case–control evidence from the British COVID Symptom Study Biobank
Source: BMJ Public Health. 2024 Feb 20;2(1):e000478. doi: 10.1136/bmjph-2023-000478 (PMC11812800; doi:10.1136/bmjph-2023-000478)
Supplement: online supplemental file 1 [file bmjph-2-1-s001.pdf]

Supplementary Table 1: Sequential binary logistic models exploring associations with non-recovery from COVID-19.

Model 1: Non-recovery from COVID-19 infection

	B	SE	Wald	Df	P	OR	95% LL	95% UL
GJH	.355	.091	15.333	1	<.001	1.426	1.194	1.703
Constant	-.845	.047	321.848	1	<.001	.430		

Model 2: Model 1 adjusted for age and sex

	B	SE	Wald	Df	P	OR	95% LL	95% UL
GJH	.287	.093	9.579	1	.002	1.332	1.111	1.598
Age	-.010	.004	6.487	1	.011	.990	.982	.998
Sex	-.226	.111	4.190	1	.041	.797	.642	.990
Constant	-.216	.231	.874	1	.350	.806		

Model 3: Model 2 adjusted for ethnic group, IMD, and education level

	B	SE	Wald	Df	P	OR	95% LL	95% UL
GJH	.287	.093	9.579	1	.002	1.332	1.111	1.598
Age	-.010	.004	6.487	1	.011	.990	.982	.998
Sex	-.226	.111	4.190	1	.041	.797	.642	.990
Ethnic group			6.235	4	.182			
Ethnic group(1)	.830	.361	5.273	1	.022	2.292	1.129	4.653
Ethnic group(2)	.358	.579	.381	1	.537	1.430	.459	4.451
Ethnic group(3)	-.505	.821	.378	1	.539	.604	.121	3.020
Ethnic group(4)	-.209	.490	.182	1	.670	.811	.311	2.119
IMD quintile			9.584	4	.048			
IMD quintile (1)	.298	.182	2.689	1	.101	1.347	.944	1.923
IMD quintile (2)	.161	.136	1.409	1	.235	1.175	.900	1.533
IMD quintile (3)	.192	.114	2.807	1	.094	1.211	.968	1.516
IMD quintile (4)	-.109	.111	.964	1	.326	.897	.722	1.114
Education level			10.802	5	.055			
Education level (1)	.694	.527	1.733	1	.188	2.001	.712	5.620
Education level (2)	.161	.226	.505	1	.477	1.174	.754	1.828
Education level (3)	.065	.214	.092	1	.761	1.067	.702	1.623
Education level (4)	-.131	.202	.422	1	.516	.877	.590	1.303
Education level (5)	-.183	.204	.807	1	.369	.832	.558	1.242
Constant	-.120	.307	.153	1	.696	.887		

GJH=Generalized Joint Hypermobility; O.R. = Odds Ratio; C.I = Confidence Interval; S.E = standard error; df = degrees of freedom; sig = significance

Supplemental Table 2: Final adjusted model exploring significant association of generalised joint hypermobility (GJH) with non-recovery from COVID-19

Model 4: Model 3 adjusted for vaccination status and vaccination number

	B	SE	Wald	Df	P	OR	95% LL	95% UL
GJH	.282	.096	8.538	1	.003	1.325	1.097	1.601
Age	-.012	.004	8.322	1	.004	.988	.980	.996
Sex(1)	-.234	.114	4.222	1	.040	.791	.633	.989
Ethnic group			6.133	4	.189			
Ethnic group (1)	.824	.361	5.199	1	.023	2.279	1.123	4.628
Ethnic group (2)	.315	.583	.292	1	.589	1.370	.437	4.291
Ethnic group (3)	-.539	.824	.428	1	.513	.583	.116	2.931
Ethnic group (4)	-.213	.490	.190	1	.663	.808	.310	2.109
IMD quintile			10.054	4	.040			
IMD quintile (1)	.297	.182	2.666	1	.103	1.346	.942	1.922
IMD quintile (2)	.174	.136	1.633	1	.201	1.190	.911	1.554
IMD quintile (3)	.196	.115	2.913	1	.088	1.216	.971	1.522
IMD quintile (4)	-.112	.111	1.027	1	.311	.894	.719	1.111
Education level			10.731	5	.057			
Education level (1)	.690	.528	1.710	1	.191	1.994	.709	5.614
Education level (2)	.177	.226	.613	1	.433	1.194	.766	1.860
Education level (3)	.054	.214	.064	1	.800	1.056	.694	1.606
Education level (4)	-.129	.202	.406	1	.524	.879	.592	1.306
Education level (5)	-.179	.204	.766	1	.381	.836	.560	1.248
Vaccination status (1)	.405	.517	.615	1	.433	1.500	.545	4.131
Vaccination number	.062	.090	.475	1	.491	1.064	.892	1.269
Constant	-.302	.389	.602	1	.438	.740		

GJH=Generalized Joint Hypermobility; O.R. = Odds Ratio; C.I = Confidence Interval; S.E = standard error; df = degrees of freedom; sig = significance
